# Combined inhibition of protein kinase B and enhancer of zeste homolog 2: a novel strategy to induce triple-negative breast cancer cell death

**DOI:** 10.1186/s43556-025-00294-3

**Published:** 2025-07-21

**Authors:** Xinxiao Zhu, Yongqiang Wang, Long Zhang

**Affiliations:** 1 Department of Radiation Oncology and the State Key Laboratory of Transvascular Implantation Devices, The Second Affiliated Hospital of Zhejiang University School of Medicine, Life Sciences Institute, Zhejiang University, Hangzhou, China; 2 The First Affiliated Hospital, the Institutes of Biology and Medical Sciences, Suzhou Medical College, Soochow University, Suzhou, 215006 China; 3 Frontiers Medical Center, Tianfu Jincheng Laboratory, Chengdu, China; 4 The MOE Basic Research and Innovation Center for the Targeted Therapeutics of Solid Tumors, The First Affiliated Hospital, Jiangxi Medical College, Nanchang University, Nanchang, China

A recent study published in *Nature* by Schade et al. [1] revealed a promising AKT (Protein Kinase B) inhibitor-based therapeutic combination for TNBC (Triple-Negative Breast Cancer). The combination of an AKT inhibitor and EZH2 (Enhancer of Zeste Homolog 2) suppressors selectively kills TNBC by exploiting mammary gland degradation mechanisms. This reveals new directions for the clinical treatment of this highly aggressive cancer type.

TNBC is a heterogeneous and clinically aggressive disease [2]. EZH2 overexpression is linked to cancer development and helps maintain tubulointerstitial progenitor cell characteristics. Previous studies have shown that AKT activation phosphorylates EZH2 at Ser21, inhibiting its methyltransferase activity. This phosphorylation causes EZH2 to dissociate from histone H3, blocking its ability to methylate H3K27 and weakening gene silencing [3]. Schade et al*.* proposed that EZH2 inhibitors (EZH2i) have the potential to enhance the sensitivity of TNBCs to AKTi by transforming poorly differentiated tumors into a more luminal-like phenotype. The researchers tested the response of several TNBC cell lines to the sequential addition of EZH2i and AKTi. The results showed that more than half of the cell lines were intolerant to this treatment, leading to a significant reduction in cell numbers in a short period. Furthermore, both AKT and EZH2 inhibitors were ineffective as single agents in killing TNBC cells. The combination treatment, however, had lasting effects and effectively inhibited cell proliferation compared to ipatasertib alone.

Next, the authors evaluated the efficacy of EZH2i in combination with AKTi in multiple tumor models. Although the effect was limited when used alone, the combination effectively induced tumor regression in five models and significantly prolonged post-treatment survival. When treated with this combination, tumor volumes in MDA-MB-468, SUM149PT, GEMM, and PDX HCl-004 xenograft models significantly decreased. Long-term treatment in the PDX HCl-004 model showed excellent results. This combination was well-tolerated in mouse models and consistently affected tumor cells, which is rare in TNBC.

Schade et al. then performed RNA-seq with treatments of either EZH2i, AKTi, or both. They showed that this combination effectively repressed genes linked to basal and mammary stem cells while upregulating luminal markers like GATA3 (GATA-binding factor 3). Gene-level bioinformatics analysis showed that treatment with EZH2/AKTi promoted the conversion of TNBC cells to a more luminal phenotype. This treatment also suppressed basal characteristics. Multiplexed cyclic immunofluorescence (CycIF) imaging further confirmed that tumors treated with both inhibitors exhibited a decrease in basal cytokeratins. They also showed an increase in luminal cytokeratins, indicating a clear shift in cell state. Additionally, transposase-accessible chromatin sequencing analysis revealed significant differences in baseline epigenetic profiles between sensitive and resistant cells. Sensitive cells exhibited open chromatin regions primarily associated with luminal and basal genes. In contrast, resistant cells showed restricted expression of mesenchymal genes. TNBC subtype and general mesenchymal traits could not reliably predict susceptibility or insensitivity to EZH2i and AKTi, predictive modeling analysis of CCLE RNA-seq data revealed that the Random Forest (RF) algorithm was effective in predicting the sensitivity of cell lines. When applying this model to transcriptomic data from TCGA Firehose TNBC tumor specimens, it predicts that 55% of the tumors are sensitive to EZH2/AKT inhibitors. This aligns with the observation that 60% of TNBC cell lines based on empirical data are actually responsive to these agents. Based on these findings, it is crucial to further investigate the potential of this signature as a predictive biomarker for treatment sensitivity.

The authors next explored the mechanisms underlying EZH2 and AKT inhibitors during the induction of GATA3. Using Chromatin conformation and Targeted sequencing to identify GATA3 enhancer sequences, the authors observed that EZH2 inhibition resulted in the loss of H3K27me3 modification. This loss occurred specifically in the GATA3 enhancer region. ATAC-seq analysis showed that EZH2 and AKT inhibitors increased the accessibility of the GATA3 enhancer. Schade et al. identified FOXO1 (Forkhead box O1) as a key regulator by screening transcription factors bound to enhancer peaks. They found that FOXO1 induction was closely linked to EZH2 and AKT inhibitors. Phosphorylation by AKT impedes the nuclear localization and transcriptional function of FOXO1 [4]. The results showed that FOXO1 dephosphorylation correlated with GATA3 induction, highlighting FOXO1's key role in cellular responses.

Besides the increased expression of tubulointerstitial markers, ssGSEA showed that the combination of EZH2 and AKT inhibitors (EZH2i + AKTi) had a synergistic effect on inducing apoptosis. In particular, BMF (Bcl-2 modifying factor), an essential inducer of apoptosis, was significantly upregulated. Inhibition of BMF blocked the cytotoxic effect in multiple cell lines. Experiments in mice showed that forced expression of EZH2 or AKT retarded degeneration, whereas their ablation accelerated it. The authors hypothesized that EZH2/AKTi promote TNBC cell death by triggering signals that promote luminal cell degeneration. They also proposed that this process upregulates BMF and other related genes via the JAK1 (Janus kinase 1)-STAT3 (Signal Transducer and Activator of Transcription 3) pathway. The synergistic effect of EZH2 and AKT inhibitors effectively induced transcriptional signatures linked to degeneration in two different cell lines. Ablation of STAT3 suppressed BMF expression.

Considering that EZH2/AKTi may work together to induce BMF upregulation and apoptosis, the authors observed that the presence of both compounds was essential for maximal JAK1–STAT3 signaling. The interleukin-6 (IL-6) cytokine family is involved in this mechanism, EZH2/AKTi significantly upregulated IL-6. Importantly, IL-6R (IL-6 receptor) was required to induce BMF expression and activate STAT3.

EZH2i are known to activate the STING (Stimulator of Interferon Genes)-TBK1 (TANK-binding kinase 1) pathway in various tumors [5]. Research findings indicate that genetic deletion of *STING* and TBK1 blocks cell death caused by EZH2/AKTi and reduces IL-6 production. Notably, EZH2i were able to induce 2′3′-cGAMP (2′3′-cyclic GMP-AMP) production alone, whereas AKTi enhanced the interaction between STING and TBK1 and promoted TBK1 activation. The authors investigated how resistant cells might be made more responsive to EZH2i and AKTi. They found activation of GATA3 and STING successfully reprogrammed non-responsive TNBC cells, making them sensitive to these inhibitors. While overexpression of GATA3 alone or STING agonists did not sensitize the cells, combining both successfully restored sensitivity to EZH2 and AKT inhibitors.

In conclusion, these studies suggest that EZH2 acts as an epigenetic insulator in TNBC. This inhibition protects tumor cells from undergoing luminal differentiation and apoptosis. AKT also plays an equally crucial role, and its inhibition enhances the upregulation of key genes, leading to cell death. While the specific ways in which EZH2 and AKT influence mouse mammary gland degeneration remain unclear, they collaboratively modulate IL-6, JAK1, STAT3, and BMF, driving cell death (Fig. 1). These findings provide strong evidence for the potential of combining AKTi and EZH2i in clinical trials for TNBC. Although the mechanisms of EZH2 and AKT in TNBC have been partially explored, the exact ways in which they synergistically activate IL-6, JAK1, STAT3, and BMF remain unclear. Further research is needed to elucidate these interactions and determine whether similar mechanisms are at play in other tumor types, with attention to potential tissue-specific differences.

**Fig. 1:**
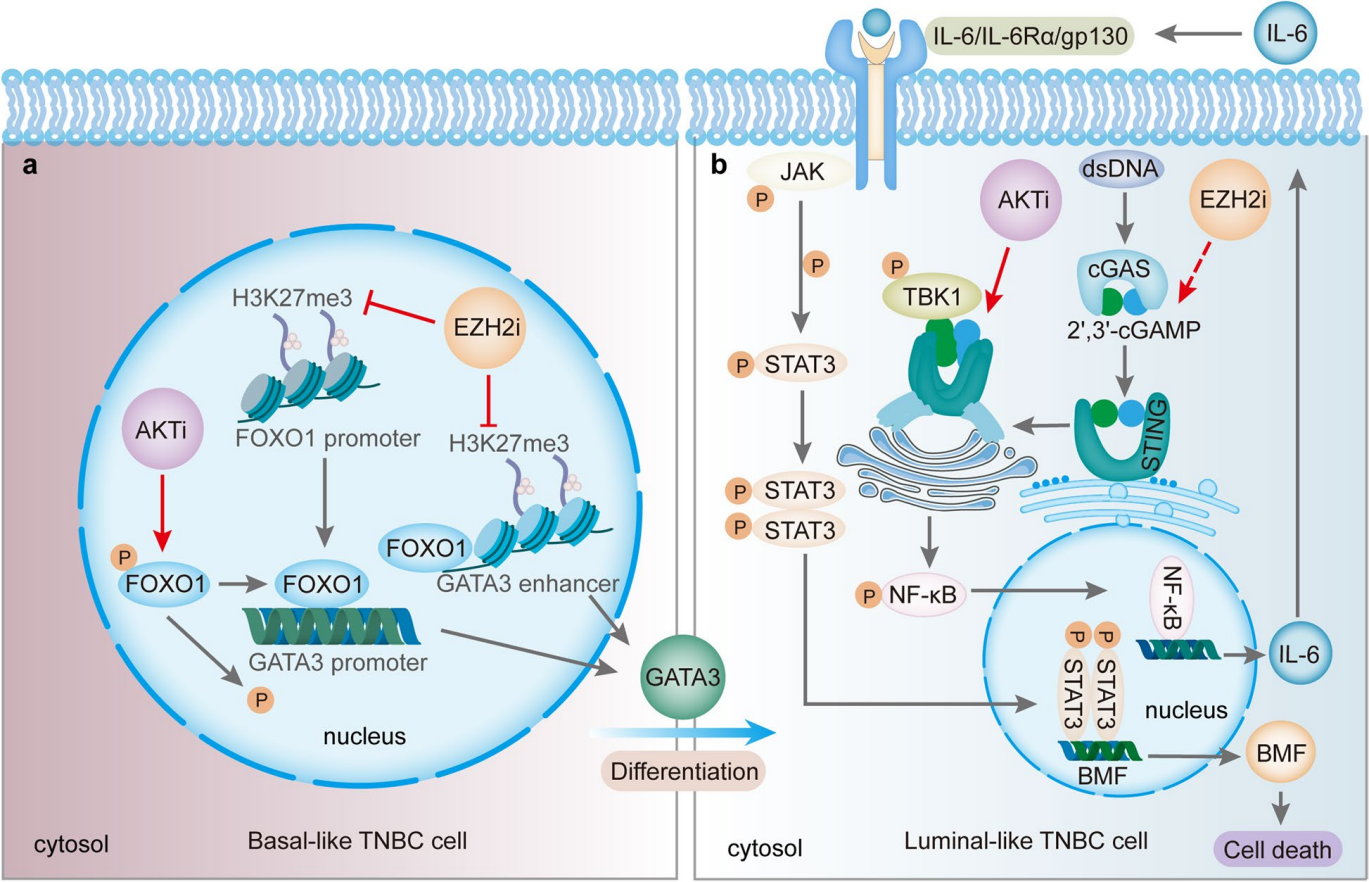
Simplified schematic of the joint activity of AKTi and EZH2i that promotes tumor regression. **a** EZH2 inhibitor (EZH2i) promotes chromatin accessibility at the GATA3 enhancer, represses H3K27me3 at the FOXO1 promoter, then induces gene expression. AKT inhibitor (AKTi) induces the removal of phosphate groups from FOXO1 at a key negative control region [4], which together induces the expression of GATA3 and puts the basal-like TNBC cells into a more differentiated tubulo-luminal-like state. **b** Subsequently EZH2i activate cGAS/2′3′-cGAMP, whereas AKTi act downstream by through the enhancement of the binding of STING and TBK1 to induce IL-6 production [5]. This in turn activates the IL-6-mediated JAK1-STAT3 signaling, that induces BMF activation and ultimately kills luminal cells. Abbreviation: TNBC, Triple-negative breast cancer; EZH2i, EZH2 inhibitor; AKTi, AKT inhibitor; H3K27me3, Trimethylation modification of lysine at position 27 of histone H3; FOXO1, Forkhead box O1; GATA3, GATA-binding factor 3; cGAS, Cyclic GMP-AMP Synthase; 2′3′-cGAMP, 2′3′-Cyclic GMP-AMP; STING; Stimulator of Interferon Genes; TBK1, TANK-binding kinase 1; IL-6, Interleukin-6; JAK1, Janus kinase 1; STAT3, Signal Transducer and Activator of Transcription 3; BMF, Bcl-2 modifying factor

## Data Availability

Not applicable.
