# Publisher Correction: Combined inhibition of protein kinase B and enhancer of zeste homolog 2: a novel strategy to induce triple-negative breast cancer cell death

**DOI:** 10.1186/s43556-025-00302-6

**Published:** 2025-08-08

**Authors:** Xinxiao Zhu, Yongqiang Wang, Long Zhang

**Affiliations:** 1 Department of Radiation Oncology and the State Key Laboratory of Transvascular Implantation Devices, The Second Affiliated Hospital of Zhejiang University School of Medicine, Life Sciences Institute, Zhejiang University, Hangzhou, China; 2 The First Affiliated Hospital, the Institutes of Biology and Medical Sciences, Suzhou Medical College, Soochow University, Suzhou, 215006 China; 3 Frontiers Medical Center, Tianfu Jincheng Laboratory, Chengdu, China; 4 The MOE Basic Research and Innovation Center for the Targeted Therapeutics of Solid Tumors, The First Affiliated Hospital, Jiangxi Medical College, Nanchang University, Nanchang, China

**Correction** **:** **Mol Biomed 6, 53 (2025)**

**https://doi.org/10.1186/s43556-025-00294-3**

Following the publication of the original article [1], it is noticed that the first author’s name contains an error due to inadvertent typesetting mistake. The first author’s name has been corrected from Xinxiao ZhuProtein to Xinxiao Zhu.

The original article [1] has been updated.
